# New insights from bidirectional Mendelian randomization: causal relationships between telomere length and mitochondrial DNA copy number in aging biomarkers

**DOI:** 10.18632/aging.205765

**Published:** 2024-04-24

**Authors:** Xinyu Yan, Peixuan Yang, Yani Li, Ting Liu, Yawen Zha, Ting Wang, Jingjing Zhang, Zhijun Feng, Minying Li

**Affiliations:** 1 Zhongshan City People’s Hospital, Xinxiang Medical University, Xinxiang 453003, Henan, China; 2 Department of Radiation Oncology, Zhongshan City People’s Hospital, Zhongshan 528403, Guangdong, China; 3 Department of Radiation Oncology, Jiangmen Central Hospital, Jiangmen 529000, Guangdong, China

**Keywords:** mitochondrial DNA copy number, causal relationship, telomere length, bidirectional two-sample Mendelian randomization

## Abstract

Mitochondrial DNA (mtDNA) copy number and telomere length (TL) are dynamic factors that have been linked to the aging process in organisms. However, the causal relationship between these variables remains uncertain. In this research, instrumental variables (IVs) related to mtDNA copy number and TL were obtained from publicly available genome-wide association studies (GWAS). Through bidirectional Mendelian randomization (MR) analysis, we examined the potential causal relationship between these factors. The forward analysis, with mtDNA copy number as the exposure and TL as the outcome, did not reveal a significant effect (*B*=-0.004, *P*>0.05). On the contrary, upon conducting a reverse analysis, it was found that there exists a positive causal relationship (*B*=0.054, *P*<0.05). Sensitivity analyses further confirmed the reliability of these results. The outcomes of this study indicate a one-way positive causal relationship, indicating that telomere shortening in the aging process may lead to a decrease in mtDNA copy number, providing new perspectives on their biological mechanisms.

## INTRODUCTION

Mitochondria, as essential organelles in cellular processes, are primarily responsible for generating the majority of cellular energy and are integral to numerous cellular functions [1, 2]. Mitochondrial DNA (mtDNA) is a critical component in the realm of biology, influencing both the quantity and functionality of mitochondria within cells [3–5]. Mitochondria possess a range of mtDNA copies, varying from several to thousands, which play a crucial role in cellular metabolic functions and energy production. Cells adjust mtDNA copy numbers in response to various physiological and pathological conditions to meet changing energy needs. Tissues with high energy demands, such as the heart [6], kidney [7], and muscles [8], typically demonstrate increased mtDNA copy numbers. Mitochondria play a vital role in regulating cellular energy homeostasis and exhibit a ‘bioenergetic reserve capacity’ that enables them to adapt to changes in metabolic requirements [9]. Conversely, in instance of compromised mitochondrial function, such as in certain pathological or physiological conditions, a decrease in mtDNA copy numbers is often observed, resulting in diminished cellular energy production [10–12]. Furthermore, it has been reported that cells respond to situations of heightened energy demand surpassing respiratory capacity by increasing mitochondrial content; however, this adaptive response may diminish with advancing age [13].

Recent advancements in the study of mtDNA copy number variation have resulted in significant discoveries. Specifically, researchers have been able to establish associations between mtDNA copy number variations and a range of diseases, such as metabolic syndrome [14–16], cardiovascular diseases [17–19], and neurodegenerative disorders [20, 21]. Furthermore, the utilization of innovative technologies like high-throughput sequencing has enhanced the precision of measuring and analyzing mtDNA copy numbers [22]. The impact of mtDNA copy number variation on the aging process has become a focal point of extensive research [23–25]. The existing findings indicate a potential decline in mtDNA replication with advancing age, potentially linked to age-related physiological alterations. In the evolving realm of personalized medicine and precision therapy, discerning individual variances in mtDNA copy number could facilitate early disease detection and the development of tailored treatment approaches. Furthermore, investigating mtDNA copy number variation as a prospective therapeutic intervention for specific conditions is poised to emerge as an innovative strategy in the future. Changes in mtDNA copy numbers have the capacity to function as both a consequence and a driver of disease advancement, encapsulating a complex biological principle that encompasses fundamental cellular processes and a wide array of clinical ramifications.

Telomeres, which are repetitive DNA sequences located at the termini of chromosomes, serve primarily to safeguard chromosomal ends from degradation and fusion, thereby preserving the integrity of genetic material [26–28]. The measurement of these sequences, referred to as telomere length (TL), is crucial for the maintenance of cellular stability and longevity [29, 30]. Owing to the nature of DNA polymerase activity, telomeres undergo gradual shortening with each round of cell division. Upon reaching a critical threshold of shortening, cells enter a state of senescence and cease proliferating, a phenomenon commonly referred to as “Hayflick’s limit” [31]. Thus, TL is recognized as a significant indicator of cellular aging and lifespan, serving as a focal point in telomere research. Recent advancements in this field have revealed the link between abnormally shortened telomeres and various diseases, including genetic disorders [32–34], cardiovascular diseases [35, 36], cancers [37–39], and aging-related illnesses [29, 40]. Additionally, lifestyle [41–43], such as alcohol consumption [44], smoking [45], stress [46], diet [47], and exercise [48], have been reported to have an impact on TL. The lengthening of telomeres is contingent upon the presence of telomerase, yet the absence of telomerase in somatic cells poses challenges in retarding the progression of cellular senescence. Conversely, the elongation of TLs may not necessarily decelerate the aging process of an organism and could potentially increase susceptibility to diseases closely associated with TL [49]. Hence, it is necessary to understand the relationship between TL and various physiological and pathological processes to enhance its predictive value in disease prognosis and individual health management. The variability in mtDNA copy number may impact the cellular energy demands required for TL maintenance [50], consequently influencing TL. Additionally, the generation of reactive oxygen species (ROS) by mitochondria during metabolic activities can directly influence TL [51, 52]. Despite some investigations into the association between TL and mtDNA, the current evidence is insufficient to definitively establish a causal relationship. Therefore, the objective of this study is to examine the possible causal association between the aforementioned aging traits through the analysis of extensive public genome-wide association study (GWAS) data, in conjunction with the application of the Mendelian randomization (MR) analysis. This methodology has the potential to provide valuable insights into elucidating the intricate interplay between TL and mitochondrial function.

## RESULTS

### The causal relationship between mtDNA copy number and TL

In the forward analysis, utilizing mtDNA as the exposure, a total of 67 instrumental variables (IVs) were identified to be associated with mtDNA, with an average *F* value of 93.32 (Supplementary Table 1). Following the exclusion of confounding factors and the matching of IVs with outcome (TL), 64 IVs remained consistent (Supplementary Table 2). Subsequently, the exposure and outcome IVs were integrated, and the “MR-PRESSO” method was employed to identify and remove outliers, resulting in 58 remaining IVs (Supplementary Table 3), of which 8 had “False” values for “mr_keep” (Supplementary Table 3). Therefore, a total of 58 IVs were included in the MR analysis for the forward analysis (Supplementary Table 3).

In the reverse analysis, TL was considered as the exposure, resulting in the identification of 154 IVs closely associated with TL, with an average *F* value of 115.74 (Supplementary Table 4). Subsequent to the exclusion of confounding factor and matching IVs with the outcome, 147 IVs were found to be identical (Supplementary Table 5). Following integration and outlier exclusion steps, 135 IVs were retained (Supplementary Table 6), among which 20 had “False” values for the “mr_keep” (Supplementary Table 6). As a result, the total number of IVs included in the MR analysis for the reverse analysis was 115 (Supplementary Table 6).

**Figure 1:**
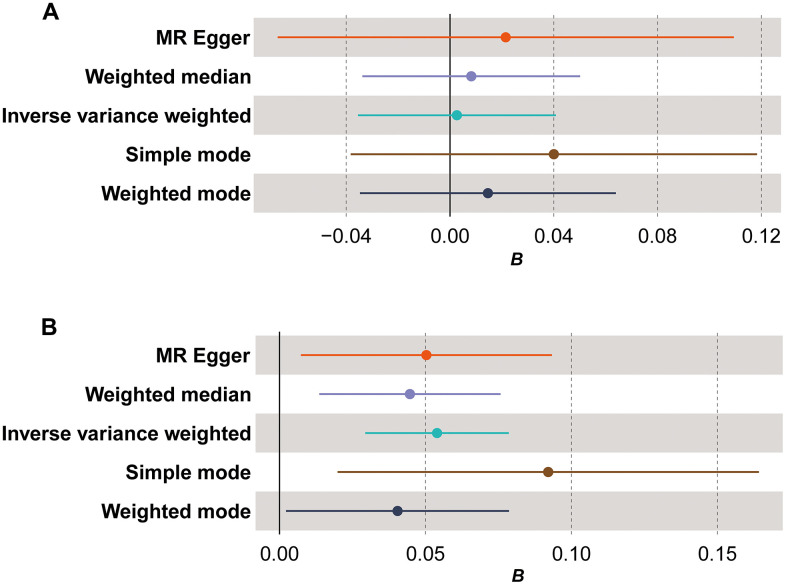
**Forest plot for Mendelian randomization (MR) results.** (**A**) The MR results for the forward analysis; (**B**) the MR results for the reverse analysis. The x-axis (*B*) shows effect estimates; dots represent primary effect estimate; horizontal lines depict the confidence interval of effect estimate.

The comprehensive findings of bidirectional MR analysis indicated that, in the forward analysis assessing the causal relationship between mtDNA copy number variation and TL, five methods (MR Egger, weighted median, inverse variance weighted (IVW), simple mode, and weighted mode) did not exhibit statistically significant results (Figure 1A, β=-0.004, *P*>0.05). Conversely, in the reverse analysis examining the causal relationship between TL and mtDNA copy number variation, all five methods consistently demonstrated a positive causal association with statistically significant results (Figure 1B, β=0.054, *P*_MR Egger_*=0.02, P*_weighted median_*=0.005, P*_IVW_*<0.0001, P*_simple mode_*=0.01, P*_weighted mode_*=0.04*). Detailed data results can be found in Supplementary Table 7. This evidence implies that alterations in TL may lead to changes in mtDNA copy number, indicating a direct causal relationship between TL and mtDNA copy number.

### Sensitivity analysis

During the sensitivity analysis, we assessed the influence of individual single nucleotide polymorphism (SNP) on the collective results of bidirectional MR analysis. Forest plots revealed variability in the effects of specific SNPs on the outcome, suggesting potential heterogeneity in current analysis (Supplementary Figures 1, 2). Nevertheless, the cumulative impact of individual SNPs on the outcome was consistent with the overarching trend observed in the MR analysis. Heterogeneity was identified in both forward and reverse analyses (Table 1, *P*<0.05); however, subsequent assessment utilizing a random-effects model yielded findings congruent with the primary MR analysis. No statistically significant variances in multiple heterogeneity were noted (Table 1, *P*>0.05), underscoring the reliability of our present analysis.

**Table 1:** Heterogeneity and pleiotropy tests of instrumental variables (IVs) in bidirectional Mendelian randomization (MR) analysis.

**Outcome**	**Exposure**	**Q *_P._*_val_**	***P*_ivw-mre_**	***P*_pleio_**
mtDNA copy number	Telomere length	2.02E-04	8.91E-01	6.43E-01
Telomere length	mtDNA copy number	8.51E-05	5.29E-05^*^	5.53E-01

mtDNA, mitochondrial DNA. *P*, *P*-value; ivw, inverse variance weightedmre; mre, random-effects model; pleio, pleiotropy test. * indicates *P*-value less than 0.05.

**Figure 2:**
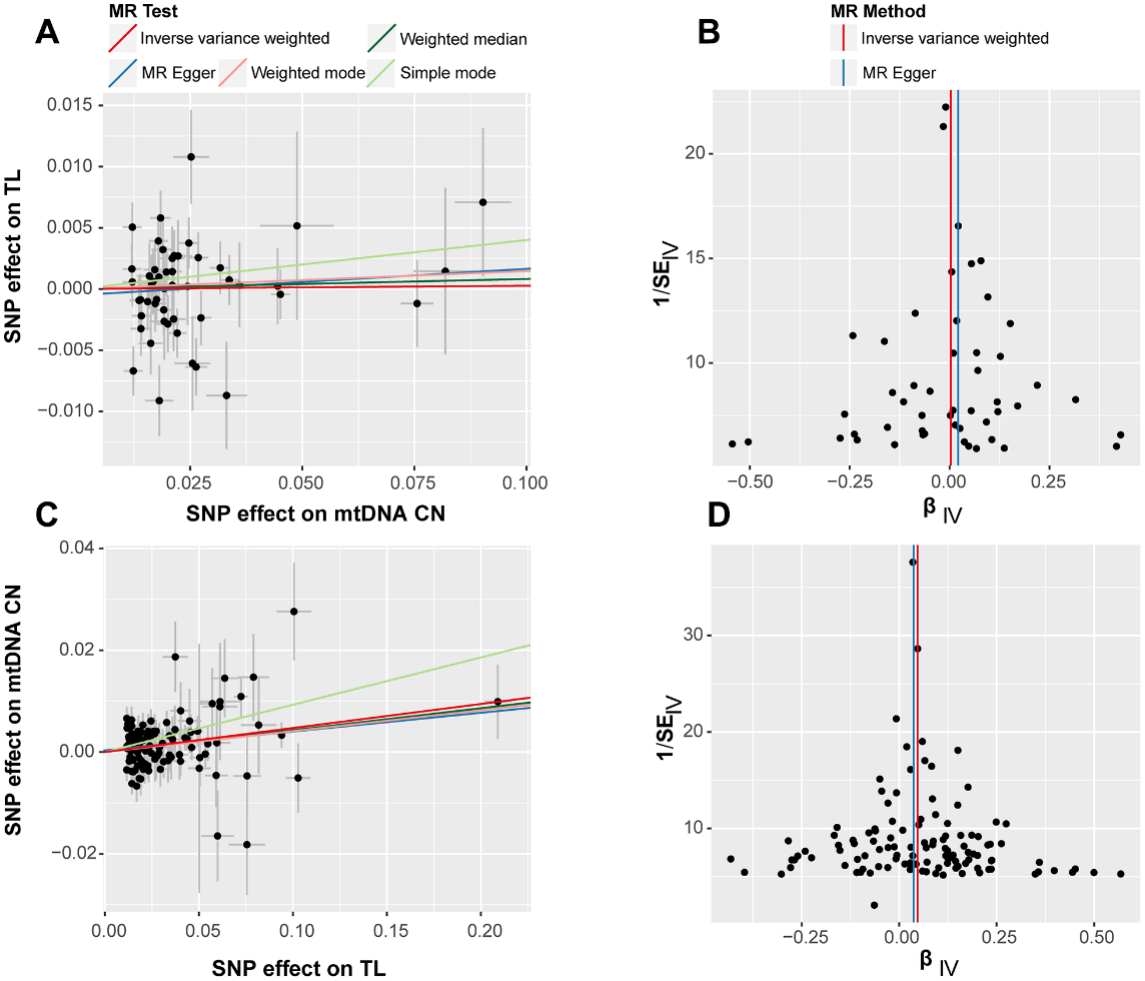
**Scatter plot (left) and funnel plot (right) for bidirectional Mendelian randomization (MR) analyses.** The results labeled (**A**, **B**) correspond to the outcomes of the forward MR analysis, while (**C**, **D**) represent the findings from the reverse MR analysis. Each point represents an included SNP in the MR analysis, and the color of the lines corresponds to the methods described in the legend.

Leave-one-out tests were conducted to evaluate the effects of excluding individual SNPs on the results of MR analysis. The findings revealed that excluding individual SNPs had minimal impact on the overall results in both forward (Supplementary Figure 3) and reverse (Supplementary Figure 4) analyses. Scatter plots showed no significant alterations in the slopes of the fitted curves representing the relationship between exposure and outcome in the forward analysis (Figure 2A), indicating a less apparent causal relationship in this direction. Conversely, in the reverse analysis (Figure 2A), the MR analysis of exposure to outcome displayed a distinct alteration in slope, suggesting a plausible causal relationship in this direction. In general, scatter plots did not demonstrate substantial deviations of outlier SNPs in any direction, indicating the lack of anomalies in the included IVs in the study.

The forest plots in bidirectional analysis, as depicted in Figure 2B for forward analysis and Figure 2D for reverse analysis, displayed a uniform distribution of individual SNPs on either side of the IVW central axis. No evidence of abnormal SNP distribution was observed. These findings highlight the reliability and robustness of the IVs included in current analysis.

**Figure 3:**
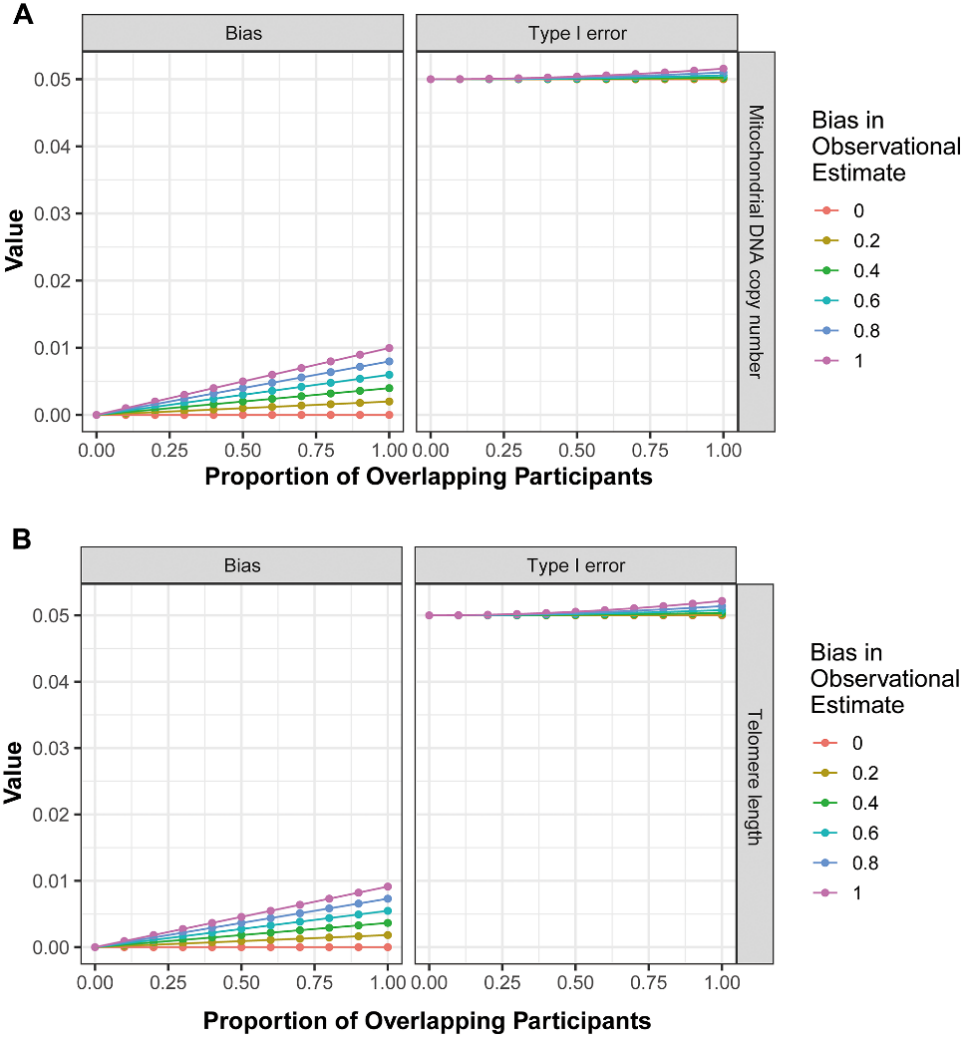
**The influence of sample overlap on the bias of MR analysis results and type I error.** (**A**) corresponds to the outcomes of the forward MR analysis, while (**B**) represents the findings from the reverse MR analysis.

Finally, the potential bias introduced by sample overlap was calculated using the ‘mrSampleOverlap’ R package. The results revealed that as the rate of sample overlap increase, there is a corresponding increase in bias and the probability of Type I errors (Figure 3A, the forward MR analysis; Figure 3B, the reverse MR analysis). However, in bidirectional analysis, this trend remained relatively stable (Figure 3), indicating that the impact of sample overlap on the results of current MR analysis is minimal.

## DISCUSSION

MR, a method in epidemiology used to evaluate causal relationships between environmental factors and diseases, employs genetic variations as IVs to control for confounding factors [53]. This methodology offers a more accurate assessment of causality, providing significant implications for personalized medicine and precision treatment due to its prospective and widely applicable nature [54, 55]. The current study utilized MR analysis to explore the causal relationship between TL and mtDNA copy number. The findings demonstrated a positive causal link between shortened TL and a decline in mtDNA copy number, suggesting that a reduction in TL is accompanied by a decrease in mtDNA copy number. In contrast, IVs associated with mtDNA copy number did not exhibit a statistically significant effect on TL. The results of sensitivity analysis supported the robustness and reliability of the findings. This evidence provides valuable insights into the complex mechanisms that involve cellular aging, enhancing our understanding of the dynamic interaction between telomeres and mitochondria in the context of cellular aging.

The regulation of TL is a complex process influenced by various factors and mechanisms. Telomeres, which are composed of DNA sequences and proteins, serve as protective structures of chromosomes [56]. During cell division, the telomeres shorten, and once the short telomere reaches a critical length, the cell stops dividing, resulting in senescence. The telomere shortening process can be reversed by telomerase; however, most somatic cells lose telomerase activity after adulthood [57, 58]. The activity of telomerase is aberrant in certain types of cells, such as stem cells and cancer cells [59, 60]. As a result, telomere lengthening not only poses a health risk, but may also increase the risk of certain diseases, including cancer [57, 61]. Thus, strategies aimed at extending telomeres to delay aging should be carefully considered. In addition, telomeres are protected by a special group of proteins called telomere protection proteins. These proteins form a unique structure that protects telomeres from degradation and damage [62]. These proteins require energy to be translated, assembled, and modified. From a physiological regulation perspective, mitochondria, which generate energy for the cell, appear to interact with TL [63].

Telomere shortening leads to aging, and aging cells undergo apoptosis (or death), damaging and degrading mtDNA, resulting in a decrease in mtDNA copy number. Thus, scholars believe mtDNA copy number decrease and TL shortening are complementary biological features of aging [64, 65]. In this study, we investigated the potential influence of IVs associated with TL on the variability of mtDNA copy number. The results indicated a positive causal relationship, suggesting that telomere shortening may lead to a reduction in mtDNA copy number. While this study did not definitively determine whether this causal relationship is direct or indirect, it did provide direct evidence of the interaction between TL and variability in mtDNA copy number. In contrast, despite the significant role of mitochondria in energy production, we did not observe any substantial causal effect of mtDNA copy number variation on TL. This result suggests that alterations in TL during cellular process are not entirely dependent on energy supply from mitochondria, a topic that warrants further exploration. Previous studies have demonstrated that an elongation of TL is associated with increased susceptibility to various malignancies, such as prostate, lung, and liver cancer [37, 66–69]. It was consistent with the findings in our analysis. The longer the telomeres, the more mtDNA copies there are, enhancing the mitochondrial function and the well-being of the body. The number of mtDNA copies is considered an important criterion for measuring mitochondrial biomass [70]. In this way, the positive causal relationship between TL and mtDNA copy number may help maintain mitochondrial health and combat aging-related diseases and processes. Long TL may contribute to increased cancer risk, as well as sufficient energy supply through mtDNA. Therefore, this is also a topic worth considering in the field of tumors [71]. These findings suggest that integrating TL and mtDNA copy number into comprehensive strategies for diagnosing and treating tumors may prove to be a novel and potentially beneficial approach. Moreover, an investigation into the regulatory mechanisms controlling TL and its association with mtDNA copy number variability offers a potential pathway for the advancement of anti-aging strategies and the improvement of cellular well-being. Exploring these intricacies will not only progress our comprehension of the fundamental biological processes but also lay the groundwork for novel interventions with extensive implications for fostering healthy aging and preventing diseases.

The results of sensitivity analysis demonstrate that this study is methodologically robust, but certain potential limitations must also be acknowledged. A notable limitation is the lack of explicit confounding factors. For example, according to correlation-based analysis, oxidative stress, free radicals, and living habits could affect TL or mtDNA copy number, but IVs related to these factors were not excluded from current MR analysis. This is because, on one hand, the evidence supporting these conclusions is derived from the analysis of public data, rather than on experimental studies or randomized trials. On the other hand, due to the lack of phenotype matching data in the GWAS database, we are unable to analyze these confounders and exclude specific IVs. Considering these potential confounding factors, we acknowledge their bias on the results of our analysis. Hence, we implemented a stricter screening criterion by requiring that the *P*-values from all five MR analysis methods be below 0.05, and there was no evidence of horizontal pleiotropy in the same direction in order to establish significant causality. Based on these rules, this MR analysis can still produce reliable results. In addition, there is a limitation to distinguish precise instances of exposure and outcome sample overlap using MR analysis. Despite application of a method to investigate the bias of sample overlap in MR analysis, the current evidences are only according to the assumptions and statistical inference, highlighting that there may be a possibility of bias in the results. The identified limitations are required to be strengthened and refined in the subsequent studies for improving the reliability of MR analysis results.

In conclusion, the current MR analysis indicates a unidirectional positive causal effect of TL on mtDNA copy number, suggesting a complex interrelationship between these two biomarkers in the aging process. This discovery provides new perspectives on the interaction between TL and mtDNA copy number and proposes novel hypotheses for their biological pathways.

## MATERIALS AND METHODS

### Study design description

**Figure 4:**
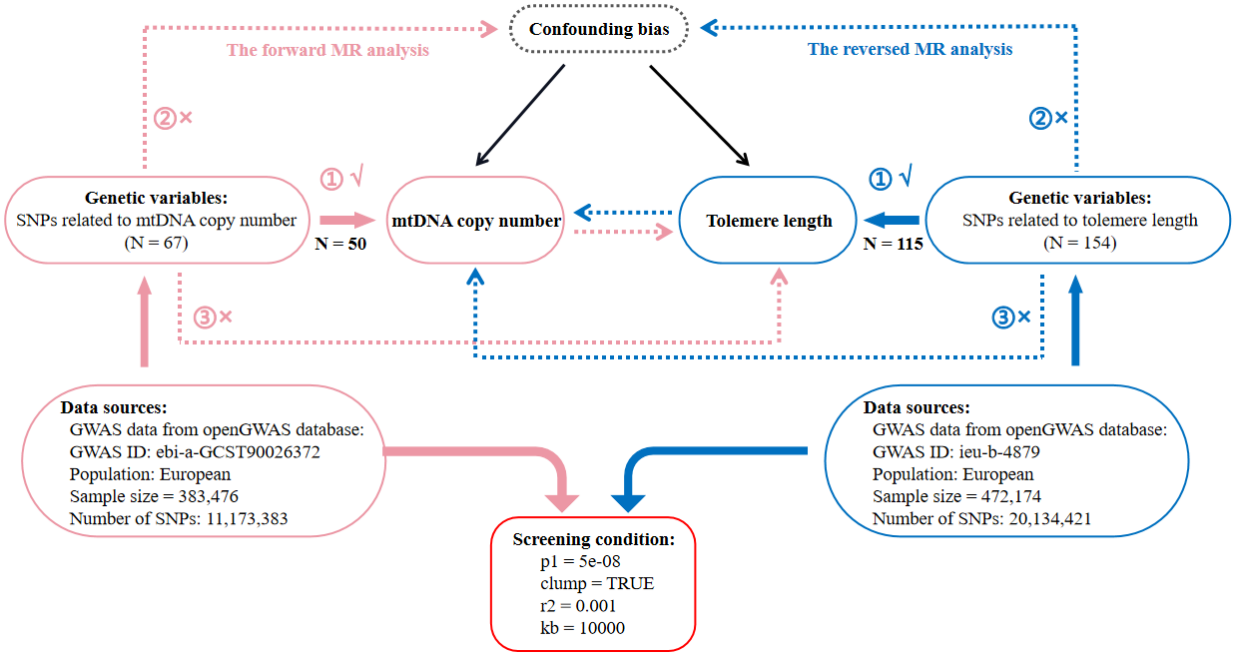
**Flow diagram of the process for the bidirectional two-sample Mendelian randomization (MR) analysis.** SNP, single nucleotide polymorphism; N, number of SNPs; mtDNA, mitochondrial DNA.

This is a two-sample MR study following STROBE-MR (Strengthening the Reporting of Mendelian Randomization Studies) guidelines [72]. Data were obtained from the openGWAS database (https://gwas.mrcieu.ac.uk/) by searching for the terms ‘telomere length’ and ‘mitochondrial DNA’. Afterwards, we selected the datasets with the largest sample size in the same race. Finally, as for TL, we used the “ieu-b-4879” [73] dataset and as for mtDNA copy number, we used the “ebi-a-GCST90026372” [5] dataset. In Figure 4, we provide the details of these candidate datasets. Using these datasets, we developed a comprehensive framework for bidirectional MR analysis to investigate the causal relationship between mtDNA copy number and TL. The forward MR analysis used mtDNA copy number as an exposure and TL as an outcome, whereas the reverse MR used TL as an exposure and mtDNA copy number as an outcome. A further explanation of the fundamental assumptions behind MR analysis can be found in Figure 4. Selected genetic variants should be examined to assess whether they satisfy the three MR assumptions: (1) IVs are strongly correlated with exposure; (2) IVs are not associated with confounding factors affecting both exposure and outcomes; (3) IVs only affect outcomes through the exposure, excluding other indirect pathways. As the data in this study were derived from publicly accessible GWAS summary statistics, no further ethical approval or informed consent was required. A detailed description of the datasets (such as GWAS ID, sample size, population category, SNP count, IV selection standards, and number of IVs) used for MR analysis can be found in Figure 4.

### IVs selection

### Identifying IVs for exposure

The ‘extract_instruments’ function from the 'TwoSampleMR' R package [74] was used to select IVs related to exposure (Figure 4). It was important to ensure that both the sample size and the effect attributable to factor (EAF) were accurate. We used openGWAS data to supplement missing sample sizes; 1000 Genomes project [75] data were used to calculate missing EAFs. After obtaining IVs, we computed F-values, ensuring that only instrumental variables with F-values greater than 10 were used in our analysis [76].

### Excluding confounders

The phenoscanner database [77] (http://www.phenoscanner.medschl.cam.ac.uk/) was utilized to exclude confounding IVs. With default settings, all SNPs associated with IVs were extracted and saved to a text file, without column names. Since TL is measured from leukocytes in peripheral blood, SNPs related to white cells or lymphocytes counts were not included, nor were SNPs related to outcome.

### Identifying outcome-related IVs

Using the ‘extract_outcome_data’ function, we obtained IVs related to outcomes. We then used the ‘harmonise_data’ function to align IVs related to exposures and outcomes. Mismatching is a common MR analysis error, especially when the number of outcome-related IVs exceeds those related to exposure. In these cases, the same effect and reference alleles must be used for each IV.

### Excluding anomalous IVs

Two primary steps were involved in this step: (1) excluding IVs whose p-values related to outcomes are less than 5e-08 [78], and (2) removing outliers using the ‘Outlier Test’ provided by the ‘MRPRESSO’ R package [79]. The Distortion Test was utilized to examine statistical differences pre and post removal of outliers.

Following these data cleansing steps, we compiled final data for MR analysis between exposure and outcomes (IVs include in MR analysis correspond to conditions where ‘mr_keep’ = TRUE).

### MR analysis

The MR analysis was performed with the ‘mr’ function from the ‘TwoSampleMR’ R package. MR Egger, weighted median, IVW, simple mode, and weighted mode methods were utilized in the analysis. Causal relationships were established based on consistent direction in effect values (β values) across all methods and statistical significance (*P*<0.05). The primary MR results were presented using forest plots.

### Sensitivity analysis

Three aspects of sensitivity analysis were performed on the MR results, including heterogeneity, pleiotropy, and leave-one-out sensitivity. By using the ‘mr_heterogeneity’ function (based on Cochran Q test), we assessed heterogeneity. A *P*_Q test_<0.05 indicated heterogeneity, thereby leading to a random effects re-evaluation of MR results [80]. By using the ‘mr_pleiotropy_test’ function (based on MR-Egger), we assessed pleiotropy. A *P*-value<0.05 suggested the presence of pleiotropy [81]. In these cases, we re-examined for anomalous IVs using the MR-PRESSO method and reanalyzed after excluding these IVs. Furthermore, we tested the reliability and robustness of IVs included in the current MR analysis by using the ‘mr_leaveoneout’ (leave-one-out sensitivity) and ‘mr_singlesnp’ functions. A scatter plot was created using the ‘mr_scatter_plot’ function to visualize trends of correlation between exposure and outcome. In addition, funnel plots generated from the ‘mr_funnel_plot’ function were used to determine the concentrations and distributions of the IVs.

### Assessing bias from sample overlap

In this study, possible biases due to varying levels of sample overlap were calculated by using the ‘estimate_overlap_bias’ function from the ‘mrSampleOverlap’ R package [82]. Estimation bias and Type I error tended to increase minimally in MR results, indicating their robustness. However, the exponential increase in bias indicates that the overlap between samples significantly impacts the current MR analysis. More MR analysis of different populations is needed to strengthen the results.

## Supplementary Material

Supplementary Figures

Supplementary Table 1

Supplementary Table 2

Supplementary Table 3

Supplementary Table 4

Supplementary Table 5

Supplementary Table 6

Supplementary Table 7
