# Supplementary material for: New insights from bidirectional Mendelian randomization: causal relationships between telomere length and mitochondrial DNA copy number in aging biomarkers
Source: Aging (Albany NY). 2024 Apr 24;16(8):7387–404. doi: 10.18632/aging.205765 (PMC11087129; doi:10.18632/aging.205765)
Supplement: Supplementary Figures [file aging-16-205765-s001.pdf]

SUPPLEMENTARY FIGURES

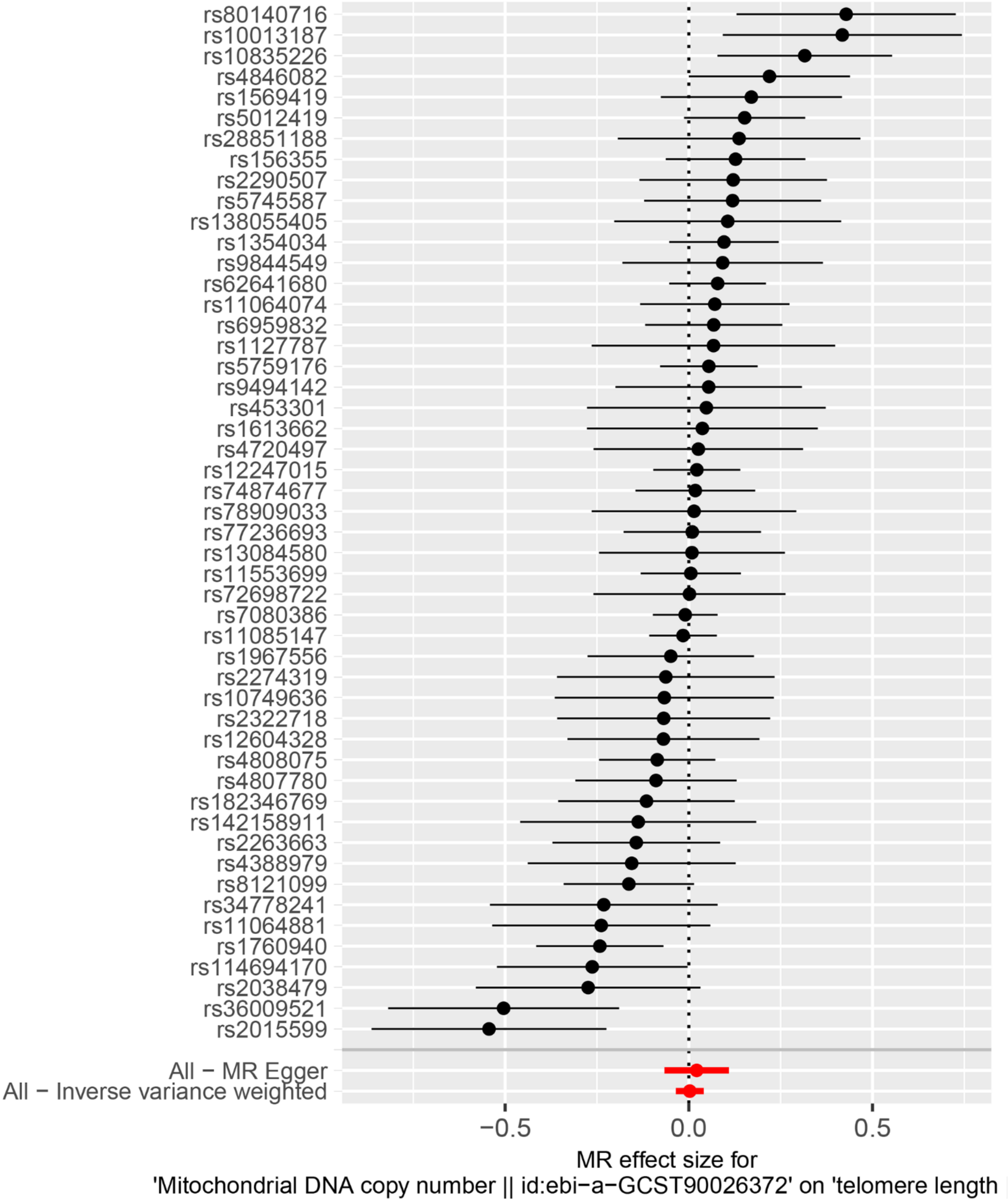

Supplementary Figure 1. Forest plot for single SNP between mtDNA copy number as exposure and TL as outcome.

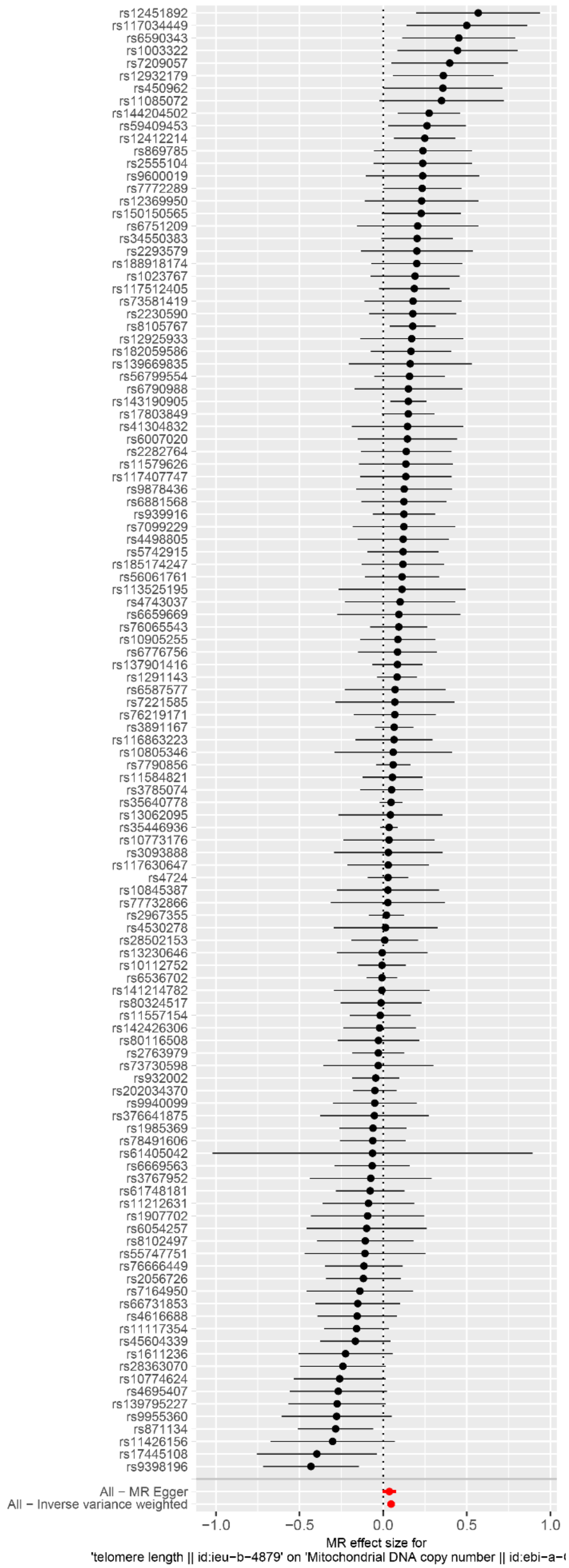

Supplementary Figure 2. Forest plot for single SNP between TL as exposure and mtDNA copy number as outcome.

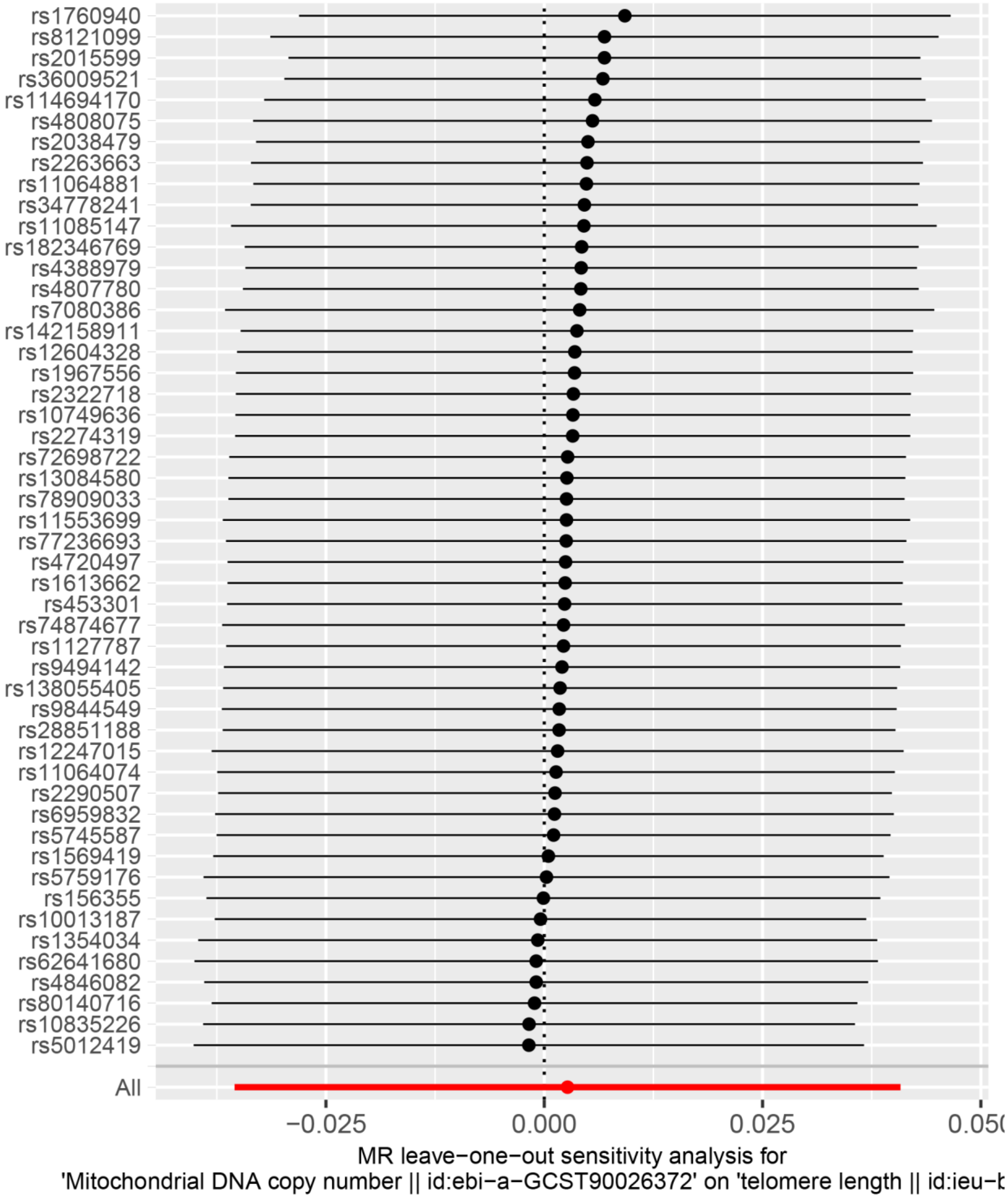

Supplementary Figure 3. Leave one out plot for MR analysis between mtDNA copy number as exposure and TL as outcome.

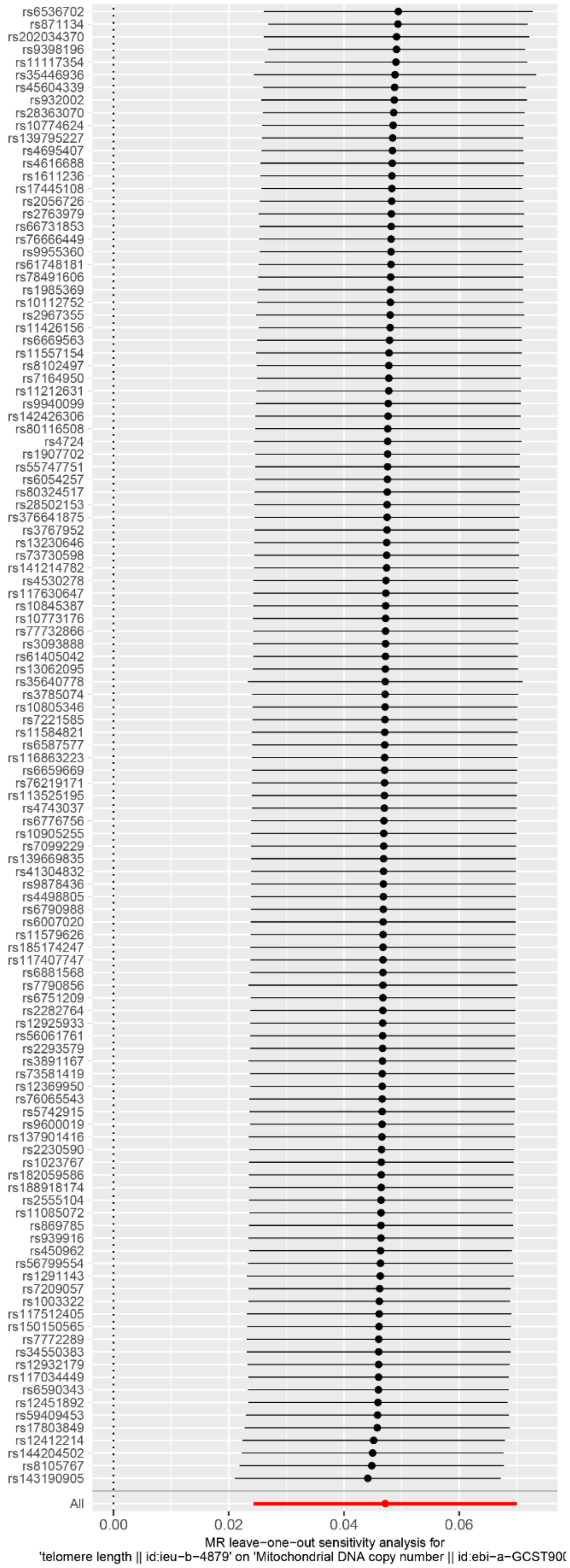

Supplementary Figure 4. Leave one out plot for MR analysis between TL as exposure and mtDNA copy number as outcome.
